# Renin-angiotensin system gene polymorphisms and high blood pressure in Lithuanian children and adolescents

**DOI:** 10.1186/s12881-017-0462-z

**Published:** 2017-09-13

**Authors:** Sandrita Simonyte, Renata Kuciene, Jurate Medzioniene, Virginija Dulskiene, Vaiva Lesauskaite

**Affiliations:** Institute of Cardiology of Medical Academy, Lithuanian University of Health Sciences, Sukilėlių 15, LT-50161 Kaunas, Lithuania

**Keywords:** AGT, Angiotensinogen; AGTR1, Angiotensin II receptor 1; ACE, Angiotensin-converting enzyme; SBP, Systolic blood pressure; DBP, Diastolic blood pressure; hypertension, Polymorphism

## Abstract

**Background:**

Epidemiological studies have demonstrated the influence of environmental factors on HBP in the population of Lithuanian children, although the role of genetic factors in hypertension has not yet been studied. The aim of this study was to assess the distribution of *AGTR1, AGT,* and *ACE* genotypes in the Lithuanian child population and to determine whether these genotypes have an impact on HBP in childhood.

**Methods:**

This cross-sectional study enrolled 709 participants aged 12–15 years. The subjects were genotyped for *AGT* (M235 T, rs699), *AGTR1* (A1166C, rs5186), and *ACE* (rs4340) gene polymorphisms using real-time and conventional polymerase chain reactions. Blood pressure and anthropometric parameters were measured.

**Results:**

The prevalence of HBP was 38.6% and was more frequently detected in boys than in girls (47.9% vs. 29.5%; *p* < 0.001). No significant differences in the frequencies of the *AGT* or *AGTR1* genotypes or alleles between boys and girls were observed, except for *ACE* genotypes. The mean SB*P* value was higher in HBP subjects with *ACE* ID genotype compared to those with *ACE* II homozygotes (*p* = 0.04). No significant differences in BP between different *AGT* and *AGTR1* genotype groups were found. Boys who carried the *ACE* ID + DD genotypes had higher odds of having HBP than carriers of the *ACE* II genotype did (controlling for the body mass index (BMI): OR_MH_ = 1.83; 95% CI, 1.11–3.02, *p* = 0.024; and controlling for waist circumference (WC): OR_MH_ = 1.76; 95% CI, 1.07–2.92, *p* = 0.035). These associations were not significant among girls. The same trend was observed in the multivariate analysis – after adjustment for BMI and WC, only boys with *ACE* ID genotype and *ACE* ID + DD genotypes had statistically significantly increased odds of HBP (aOR = 2.05; 95% CI, 1.19–3.53 (*p* = 0.01) and aOR = 1.82; 95% CI, 1.09–3.04 (*p* = 0.022), respectively).

**Conclusions:**

The evaluated polymorphisms of the *AGT* and *AGTR1* genes did not contribute to the presence of HBP in the present study and may be seen as predisposing factors, while *ACE* ID genotypes were associated with significantly increased odds for the development of HBP in the Lithuanian child and adolescent population - especially in boys.

## Background

Recent epidemiological studies have shown that the prevalence of high blood pressure or hypertension has significantly increased among children and adolescents worldwide [1–3]. Hypertension is one of the major cardiovascular risk factors and is associated with cardiovascular mortality, and cardiovascular diseases are the main cause of death in Lithuania [4, 5]. In Lithuania, the prevalence of increased BP or hypertension in children and adolescents is 21% [6] and 35% [3], respectively. In the adult population, approximately 60% of men and 45% of women aged 25–64 years have elevated BP [7]. High blood pressure in childhood commonly leads to hypertension in adulthood [8–10], which is the leading cause of premature death worldwide [11]. Hypertension in children can lead to left ventricular hypertrophy and pathologic vascular changes [12, 13].

Studies support the fact that hypertension is a complex disease resulting from the interactions of genes and environmental factors [14]. Epidemiological studies suggest that genetic factors account for approximately 30% of the variation in blood pressure [15–17]. Studies have demonstrated that hypertension is twice as common in subjects who have one or two hypertensive parents [18, 19]. Twin studies have also shown that genes could explain approximately 50% of blood pressure variation [20].

The renin-angiotensin-aldosterone system (RAAS) is a major regulator of arterial pressure and as an important endocrine and paracrine system plays a key role in the pathogenesis of essential hypertension [21]. Investigations have shown that the genetic variations of the RAAS-encoding genes have strong connections with the genetic basis of essential hypertension and antihypertensive treatment [22]. An increased risk of hypertension has been associated with angiotensinogen (*AGT*), angiotensin-converting enzyme (*ACE*), and angiotensin II receptor 1 (*AGTR1*). Angiotensinogen is a protein secreted by the liver and found in the α2- globulin fraction of plasma. The enzyme renin cleaves AGT forming angiotensin I, and ACE or kinase II as dipeptidyl carboxypeptidase encoded by the *ACE* gene produces the active hormone angiotensin II that promotes vasoconstriction. The M235 T (methionine substituted by threonine) polymorphism in the *AGT* gene was associated with hypertension in Caucasians and with a 10–20% increase in plasma angiotensinogen levels in individuals homozygous for the T allele [23]. ACE plays an important role in blood pressure regulation and electrolyte balance by hydrolyzing angiotensin I into angiotensin II. ACE is not only a membrane-bound enzyme on the surface of the vascular endothelial cells, but it also circulates in blood plasma [24]. The *ACE* insertion/deletion (I/D) polymorphism is associated with the concentration of the circulating enzyme. Individuals homozygous for the D allele have higher tissue and plasma ACE concentrations than heterozygotes and II homozygotes do [25]. Angiotensin 2 receptor type 1 encoded by *AGTR1* gene mediates the major cardiovascular effects of angiotensin II, leading to such effects as vasoconstriction, increased arterial blood pressure, and myocardial contractility [26]. Studies have shown that A/C transversion at position 1166 in the 3′ untranslated region of the *AGTR1* gene could be associated with hypertension [27–29].

Although relationships between overweight and obesity with prehypertension and hypertension have been established [3], the association between polymorphisms in RAS genes and HBP among Lithuanian schoolchildren based on obesity-related traits has not been studied yet.

The aim of the present cross-sectional study was to investigate if polymorphisms of the RAS genes *AGT, AGTR1,* and *ACE* might be associated with an increased risk for higher blood pressure and hypertension in the Lithuanian children population.

## Methods

### Study design and population

This cross-sectional study included a randomly selected sample of 709 subjects aged 12–15 years, who had participated in the baseline survey of “Prevalence and risk factors of high blood pressure in 12–15-year-old Lithuanian children and adolescents (Study 1, 2010-2012)” in Kaunas city and Kaunas district, located in Kaunas County, Lithuania [3]. The school children who had congenital heart defects, kidney diseases, or endocrine diseases were excluded from the study.

The sample size was estimated using the formula n = z^2^pq/d^2^, where: n – sample size, z = 1.96 at the 95% confidence level, *p* = 0.35 (the prevalence of HBP was 35% among Lithuanian adolescents aged 12–15 years [3]), q = 1–p and d = 0.05 (the degree of precision). The minimum sample size was calculated to be 350 participants.

### Measurements

Blood pressure and anthropometric measurements (weight, height, and waist circumference (WC)) performed in this study have been presented in our previous publication [3].

#### Blood pressure measurement

BP measurements were performed with an automatic BP monitor using a cuff of an appropriate size. The average of three BP measurements was calculated and used in the analysis, according to “The 4^th^ Report on the Diagnosis, Evaluation, and Treatment of High Blood Pressure in Children and Adolescents” (National High Blood Pressure Education Program (NHBPEP) Working Group on High Blood Pressure in Children and Adolescents) [30]. High BP was defined as systolic blood pressure (SBP) and diastolic blood pressure (DBP) ≥90th percentile.

#### Anthropometric measurement

All subjects underwent anthropometric measurements. According to the cut-off points of the BMI for children and adolescents proposed by the International Obesity Task Force (IOTF) [31], the subjects were grouped into three categories: normal-weight, overweight, and obese. Using the cut-off values of the percentiles of the WC according to the criteria of the Third National Health and Nutrition Examination Survey (NHANES III) [32], the participants were classified into three groups on the basis of their WC: below the 75th percentile, 75th < 90th percentile, and ≥90th percentile. High waist value was defined as WC ≥75th percentile.

### Genetic analysis

For DNA extraction, saliva samples were collected into tubes from each individual. Genomic DNA was extracted using a commercial DNA isolation kit - “Genomic DNA Purification Kit” (“Thermo Fisher Scientific”, Lithuania) according to the manufacturer’s instructions. Aliquots of purified DNA were stored at −20 °C until use in genetic analysis.

Subjects were genotyped for *AGT* (M235 T, rs699) and *AGTR1* (A1166C, rs5186) gene polymorphisms with TaqMan allelic discrimination Assay-By-Design genotyping kits: C_1985481_20 and C_3187716_10, respectively (Applied Biosystems, Foster City, CA, USA).

To analyze *ACE* (ID, rs4340) polymorphism, genomic DNA was amplified by PCR reaction with primers 5′-AGGAGAGGAGAGAGACTCAAGCACG-3′ and 5′-GGCAGC CTGGTTGATGAGTTCC-3′ [33]. PCR was performed in a 25-μL volume containing approximately 50 ng of the genomic template, 0.4 μmol/L of each primers, 200 μmol/L of each deoxynucleotide triphosphate (dNTP) (Termofisher, Lithuania), 1 unit of Maxima Hot Start *Taq* polymerase (Termofisher, Lithuania), 2 mmol/L of MgCl_2_ (Termofisher, Lithuania), and 1X Maxima Hot Start PCR Buffer (Termofisher, Lithuania). Cycling conditions were preceded by a denaturing step at 95 °C for 10 min, followed by 32 cycles of denaturation at 94 °C for 30 s, annealing at 68.8 °C for 30 s, and synthesis at 72 °C for 1 min and then at 59 °C for 1 min. The reaction was ended by incubation at 72 °C for 10 min. PCR products were separated by electrophoresis (100 V for 1.5 h; 1.5% agarose gel) and visualized by ethidium bromide-stained agarose gel under ultraviolet light using a video documentation system, the BioDocAnalyse 2.0 (Biometra, Göttingen, Germany). The I allele of *ACE* manifested as a 700 bp band, and the D allele was seen as a 400 bp band of DNA. Each *ACE* DD genotype was confirmed through a second PCR with primers specific for the insertion sequence as previously described [33].

DNA sequencing for different *AGT*, *AGTR1,* and *ACE* gene genotypes was used for quality control.

### Statistical analysis

Continuous variables were expressed as mean ± standard deviation (SD). The normality of the distribution of continuous variables was assessed by the Kolmogorov-Smirnov test. Comparisons between the groups were performed by applying the chi-square (χ^2^) test and Student’s t-test. The χ^2^ test was used for the assessment of the Hardy-Weinberg equilibrium (HWE) for the distribution of genotypes. Logistic regression analyses were used to test for the associations of *AGT*, *AGTR1,* and *ACE* polymorphisms with HBP under inheritance models to calculate the odds ratios (ORs, 95% CI) for *AGT, AGTR1,* and *ACE* polymorphism. ORs were adjusted for BMI and WC. The Mantel-Haenszel technique was used to estimate the associations of *AGT*, *AGTR1,* and *ACE* polymorphisms with HBP while controlling for BMI and WC separately. The data were analyzed separately for boys and girls.

## Results

The characteristics of the study subjects according to BP levels are presented in Table 1. Among 709 study subjects aged 12–15 years (mean ± SD age: 13.27 ± 1.14 years), 49.8% (*n* = 353) were boys and 50.2% (*n* = 356) were girls. There was no significant difference in the mean age between these groups.

**Table 1:** Characteristics of the study population according to the BP level

Characteristics	HBP (*n* = 274)	NBP (*n* = 435)	*P* value
Age, years, mean (SD)	13.30 (1.13)	13.26 (1.15)	0.647
SBP, mm Hg, mean (SD)	137.03 (10.89)	110.94 (7.35)	<0.001
DBP, mm Hg, mean (SD)	70.17 (8.20)	62.25 (6.59)	<0.001
BMI, kg/m^2^, mean (SD)	21.31 (3.54)	18.68 (2.81)	<0.001
WC, cm, mean (SD)	72.27 (9.05)	64.58 (6.89)	<0.001
Weight, kg, mean (SD)	58.71 (12.41)	49.26 (10.77)	<0.001
Height, cm, mean (SD)	165.48 (8.82)	161.67 (9.65)	<0.001
Sex, n (%)			
Boys	169 (47.9)	184 (52.1)	<0.001
Girls	105 (29.5)	251 (70.5)	
Age, years, n (%)			
12–13	156 (36.9)	267 (63.1)	0.240
14–15	118 (41.3)	168 (58.7)	
BMI categories, n (%)			
Normal weight	195 (33.0)	396 (67.0)	<0.001
Overweight	58 (62.4)	35 (37.6)	
Obesity	21 (84.0)	4 (16.0)	
WC percentile categories, n (%)			
< 75th	204 (33.2)	411 (66.8)	<0.001
75th–<90th	48 (69.6)	21 (30.4)	
≥ 90th	21 (87.5)	3 (12.5)	

The means were compared using the *t* test

Categorical variables were compared using the chi-square (χ^2^) test

*Abbreviations*: *NBP* normal blood pressure, *HBP* high blood pressure, *SBP* systolic blood pressure, *DBP* diastolic blood pressure, *BMI* body mass index, *WC* waist circumference, *SD* standard deviation

The overall prevalence of HBP was 38.6% in the entire study sample. HBP was more frequently detected in boys than in girls (47.9% vs. 29.5%; *p* < 0.001). Boys aged 14 to 15 years were more likely to have HBP than boys aged 12 to 13 years (56.8% vs. 41.5%; *p* = 0.005). Higher mean values of SBP and WC were detected in boys with HBP (SBP: 139.47 mmHg; WC: 73.21 cm) than in girls with HBP (SBP: 133.11 mmHg; WC: 70.77 cm), p < 0.001 and *p* = 0.024, respectively (data not shown). Boys with HBP had significantly higher SBP. The participants with HBP had significantly higher mean values of BMI, WC, weight, and height, compared to normotensive participants. Overweight, obesity, and high WC (**≥**75th percentile) were much more common in subjects with HBP than in the normal blood pressure (NBP) group for both sexes separately.

Allele and genotype frequencies in HBP and NBP groups were in the Hardy-Weinberg equilibrium (*p* > 0.05) (Table 2). No significant differences in the frequency of the *AGT* or *AGTR1* genotypes or alleles were observed between normotensive subjects and those with HBP according to sex, except for *ACE* genotypes in boys (*p* = 0.021).

**Table 2:** Distribution of the *AGT, AGTR1,* and *ACE* genotypes and the frequency of alleles in the study population

Characteristics	Boys		Girls	
	HBP	NBP	HBP	NBP
*AGT*, M235 T genotypes, n (%)				
MM	45 (27.3)	49 (27.1)	28 (27.2)	65 (26.7)
MT	82 (49.7)	77 (42.5)	57 (55.3)	125 (51.2)
TT	38 (23.0)	55 (30.4)	18 (17.5)	54 (22.1)
	χ^2^ = 2.70, *p* = 0.259		χ^2^ = 0.99, *p* = 0.607	
*AGT*, M235 T alleles, frequency				
M	0.52	0.48	0.55	0.52
T	0.48	0.52	0.45	0.48
	χ^2^ = 0.99, *p* = 0.321		χ^2^ = 0.39, *p* = 0.531	
*AGTR1*, A1166C genotypes, n (%)				
AA	87 (52.4)	85 (47.2)	59 (56.2)	123 (50.2)
AC	67 (40.4)	77 (42.8)	39 (37.1)	107 (43.7)
CC	12 (7.2)	18 (10.0)	7 (6.7)	15 (6.1)
	χ^2^ = 1.35, *p* = 0.508		χ^2^ = 1.29, *p* = 0.524	
*AGTR1*, A1166C alleles, frequency				
A	0.73	0.69	0.75	0.72
C	0.27	0.31	0.25	0.28
	χ^2^ = 1.32, *p* = 0.251		χ^2^ = 0.55, *p* = 0.450	
*ACE*, ID genotypes, n (%)				
II	34 (20.6)	58 (31.6)	25 (24.0)	65 (26.4)
ID	94 (57.0)	79 (42.9)	55 (52.9)	126 (51.2)
DD	37 (22.4)	47 (25.5)	24 (23.1)	55 (22.4)
	χ^2^ = 7.74, p = 0.021		χ^2^ = 0.22, *p* = 0.897	
*ACE,* ID alleles, frequency				
I	0.49	0.53	0.50	0.52
D	0.51	0.47	0.50	0.48
	χ^2^ = 1.06, *p* = 0.304		χ^2^ = 0.14, *p* = 0.707	

Comparisons between groups were performed by applying the χ^2^ test

*Abbreviations*: *NBP* normal blood pressure, *HBP* high blood pressure, *AGT* angiotensinogen, *AGTR1* angiotensin II receptor 1, *ACE* angiotensin-converting enzyme

Although obesity-related traits such as BMI and WC did not differ according to *AGT*, *AGTR1,* or *ACE* genotypes between HBP and NBP subjects, overweight combined with obesity were much more common in boys with the *ACE* ID + DD genotype compared to *ACE* II carriers with high SBP (*p* = 0.04) (data not shown).

The mean SBP and DBP levels of the participants according to genotypes are shown in Table 3. The mean values of SBP were significantly higher in boys with HBP and the *ACE* ID and *AGTR1* AC genotypes compared to the *ACE* II and *AGTR1* AA carriers. No association was observed in the group of girls.

**Table 3:** Distribution of *AGT* M235 T, *AGTR1* A1166C, and *ACE* ID genotypes according to systolic and diastolic blood pressure in the study population

Characteristics	HBP		NBP	
	SBP (mmHg)	DBP (mmHg)	SBP (mmHg)	DBP (mmHg)
Boys				
*AGT*, M235 T genotypes, mean (SD)				
MM	139.56 (10.69)	68.35 (8.16)	112.57 (6.62)	62.65 (7.61)
MT	138.72 (10.92)	69.07 (8.47)	111.65 (8.50)	61.03 (7.25)
TT	139.32 (11.94)	67.11 (7.04)	109.71 (7.68)	61.41 (6.47)
*AGTR1*, A1166C genotypes, mean (SD)				
AA	138.30 (9.82)	69.15 (7.98)	109.87 (7.95)	61.25 (7.23)
AC	139.77 (11.76)^a^	67.82 (8.18)	112.74 (7.58)	62.11 (7.24)
CC	141.11 (14.54)	66.19 (7.29)	111.15 (7.34)	61.59 (5.62)
*ACE*, ID genotypes, mean (SD)				
II	135.33 (8.22)	68.55 (7.69)	110.96 (7.39)	62.45 (8.03)
ID	139.90 (12.28)^b^	68.55 (8.33)	110.38 (8.71)	60.87 (6.74)
DD	140.21 (8.99)	67.65 (7.65)	112.56 (7.05)	61.40 (6.95)
Girls				
*AGT*, M235 T genotypes, mean (SD)				
MM	133.20 (10.21)	73.64 (8.43)	111.38 (7.27)	63.73 (5.19)
MT	133.26 (9.31)	73.16 (7.11)	110.60 (6.31)	62.24 (6.22)
TT	131.56 (6.06)	70.72 (8.63)	110.08 (8.10)	62.96 (6.70)
*AGTR1*, A1166C genotypes, mean (SD)				
AA	133.49 (9.62)	72.13 (7.36)	110.31 (6.86)	63.09 (6.38)
AC	133.44 (8.59)	74.21 (7.87)	111.03 (7.35)	62.49 (5.98)
CC	128.00 (5.47)	71.95 (9.77)	111.44 (4.78)	62.62 (4.14)
*ACE*, ID genotypes, mean (SD)				
II	132.72 (7.49)	71.89 (7.55)	111.45 (7.88)	62.69 (7.01)
ID	133.39 (9.47)	72.42 (7.29)	110.83 (6.25)	62.75 (5.36)
DD	132.49 (9.89)	74.54 (8.65)	109.58 (7.30)	62.97 (6.51)

*Abbreviations*: *NBP* normal blood pressure, *HBP* high blood pressure, *SBP* systolic blood pressure, *DBP* diastolic blood pressure, *AGT* angiotensinogen, *AGTR1* angiotensin II receptor 1, *ACE* angiotensin-converting enzyme

^a^ *p* < 0.05 between *AGTR1* AC vs. *AGTR1* AA in boys with HBP (*t* test)

^b^ *p* < 0.05 between *ACE* ID vs. *ACE* II in boys with HBP (*t* test)

The results of multivariate logistic regression analyses are presented in Table 4. To examine sex-specific associations with HBP, we calculated adjusted odds ratios by BMI and WC for boys and girls separately. No significant association between *AGT* and *AGTR1* genotypes and HBP was found in either boys or girls.

**Table 4:** Associations of AGT, AGTR1, and ACE polymorphisms with high blood pressure by sex (multivariate analysis)

		Boys		Girls	
Model	Genotypes	aOR^a^ (95% CI)	*P* value	aOR^a^ (95% CI)	*P* value
	AGT				
Co-dominant	MM	1.00		1.00	
	MT	1.20 (0.70–2.03)	0.509	1.03 (0.58–1.84)	0.926
	TT	0.72 (0.39–1.33)	0.296	0.72 (0.34–1.54)	0.400
Dominant	MM	1.00		1.00	
	MT + TT	0.99 (0.61–1.64)	0.994	0.94 (0.54–1.63)	0.817
Recessive	MM + MT	1.00		1.00	
	TT	0.65 (0.39–1.07)	0.091	0.71 (0.37–1.36)	0.301
Additive		0.85 (0.63–1.15)	0.304	0.87 (0.60–1.25)	0.448
	AGTR1				
Co-dominant	AA	1.00		1.00	
	AC	0.84 (0.53–1.33)	0.451	0.99 (0.59–1.68)	0.986
	CC	0.66 (0.29–1.48)	0.309	1.21 (0.44–3.33)	0.771
Dominant	AA	1.00		1.00	
	AC + CC	0.80 (0.52–1.24)	0.323	1.02 (0.62–1.69)	0.929
Recessive	AA + AC	1.00		1.00	
	CC	0.71 (0.32–1.56)	0.391	1.21 (0.45–3.25)	0.700
Additive		0.82 (0.58–1.15)	0.256	1.05 (0.70–1.57)	0.821
	ACE				
Co-dominant	II	1.00		1.00	
	ID	**2.05 (1.19–3.53)**	0.010	0.92 (0.51–1.67)	0.793
	DD	1.43 (0.76–2.69)	0.226	0.89 (0.44–1.82)	0.749
Dominant	II	1.00		1.00	
	ID + DD	**1.82 (1.09–3.04)**	0.022	0.91 (0.52–1.60)	0.752
Recessive	II + ID	1.00		1.00	
	DD	0.89 (0.53–1.48)	0.646	0.94 (0.52–1.70)	0.834
Additive		1.20 (0.88–1.63)	0.253	0.94 (0.66–1.35)	0.745

*Abbreviations*: *AGT* angiotensinogen, *AGTR1* angiotensin II receptor 1, *ACE* angiotensin-converting enzyme, *aOR* adjusted odds ratio, *CI* confidence intervals

^a^adjusted for the body mass index and waist circumference

The results showed that boys who carried the *ACE* ID genotype and the *ACE* ID + DD genotypes had higher odds of having HBP than carriers of the *ACE* II genotype did (in the co-dominant model, aOR = 2.05; *p* = 0.01, and in the dominant model, aOR = 1.82; *p* = 0.022, respectively).

Boys who carried the *ACE* ID + DD genotypes had higher odds of having HBP than carriers of the *ACE* II genotype did (controlling for BMI: OR_MH_ = 1.83; 95% CI, 1.11–3.02, *p* = 0.024; and controlling for WC: OR_MH_ = 1.76; 95% CI, 1.07–2.92, *p* = 0.035). These associations were not significant among girls (data not shown).

## Discussion

To our knowledge, this is the first cross-sectional study investigating the association between polymorphisms in RAS genes and hypertension among 12–15 year-old Lithuanian schoolchildren based on obesity-related traits.

Studies on elevated blood pressure or hypertension among children and adolescents showed that the prevalence rates varied widely [34–36]. According to our data, the prevalence of high blood pressure was 38.6%, and was more frequently detected in boys than in girls.

The prevalence of overweight is high among children across Europe, with an especially worrisome prevalence in Southern European countries [37]*.* A study in Lithuania showed that the prevalence of overweight and obesity among 7–17 year-old children and adolescents was 12.6% and 4.1%, respectively, with a higher prevalence among boys [38]. The prevalence of overweight and obesity in our study on 12–15 year-old children was similar (13.1% and 3.5%, respectively), but was inconsistent with the results of other studies conducted in the Czech Republic [39] and in Spain [40], which reported higher prevalence rates of overweight or obesity. As compared to the data published in other countries, Lithuania still has the lowest prevalence of overweight and obesity among 7–17 year-old children and adolescents in Europe, being similar to Poland [41] and Latvia [37] in this respect. The present study and other epidemiological studies indicate that the prevalence of HBP is higher among overweight and obese children compared to children with normal weight [42, 43]. Studies have shown that overweight, obesity, and high waist circumference were significantly associated with hypertension or HBP (≥90th percentile) among children and adolescents [44–46]. Patients with HBP had significantly higher mean values of the body mass index and waist circumference compared to normotensive children for both sexes separately. Higher systolic blood pressure and waist circumference were more commonly observed in boys than in girls.

The RAS system includes multiple components and plays a key role in BP regulation [21]. The enzyme renin cleaves the substrate AGT to produce angiotensin I (Ang I). Ang I is converted to Ang II by ACE, an enzyme present on the cell surface of many cells, mainly on vascular endothelial cells. Ang II stimulates renal sodium reabsorption and vasoconstriction, and thus raises blood pressure. Most of hypertensinogenic actions of Ang II go through specific angiotensin-receptors such as AGTR1. AGTR2 receptors promote vasodilatation, cell differentiation, and the inhibition of cell growth and apoptosis [22]. Mutations in the genes of the RAS components might be associated with the pathogenesis of hypertension. The Jeunemaitre group was the first to report the linkage of the molecular variants M235 T with hypertension in Caucasians [23]. The 235 T allele of the *AGT* gene was shown to be linked to an increased level of circulating angiotensinogen [23, 47, 48]. The level of circulating ACE depends on *ACE* gene (I/D) polymorphism. Persons with the D allele have higher plasma ACE levels and higher rates of hypertension, compared to carriers with the I allele [23, 25]. Studies found that subjects with the ID and DD genotype of the *ACE* I/D gene polymorphism had a higher increase in both pulse and SBP when compared to subjects with the II genotype [49].

In our study, the frequencies of *AGT*, *AGTR1,* and *ACE* genotypes and alleles were similar to those reported in other Caucasian populations [50, 51]. According to multivariate logistic regression analysis of our data, the present study failed to demonstrate that *AGT* M235 T or *AGTR1* A1166C polymorphisms had any association with hypertension among either boys or girls, except for the *ACE* ID polymorphism. An insertion/deletion (I/D) polymorphism of 287 base pairs is one of the most intensively investigated polymorphisms in the field of research on hypertension and cardiovascular diseases [19, 25, 52]. The present study identified a sex-specific variant in the *ACE* gene and its link to elevated blood pressure. We found significant evidence of an association between the *ACE* ID genotype and elevated systolic blood pressure in boys, but not in girls. Increased odds for hypertension in the boys’ population were higher with *ACE* ID + DD than with *ACE* II genotypes. In boys, the dominant model of the *ACE* gene even after adjustment for the body mass index and waist circumference (ID + DD versus II) showed that D allele carriers were by 1.82 times more likely to be hypertensive than carriers of the II genotype. Our results are generally consistent with the majority of other studies conducted in child and adult populations [9, 51, 53–55]. The mechanism of the sex-specific association with high blood pressure or hypertension remains unclear. There is an opinion that estrogens could play a protective role against hypertension by regulating the RAS, mainly through the angiotensin-converting enzyme 2/Ang(1–7)/Mas receptor (MasR) and AT_2_R pathways [56, 57].

The polymorphism A1166C in the 3′ untranslated region of the *AGTR1* gene was detected in a study by Bonnardeaux et al. (1994) who also identified its association with hypertension [58]. Several recent findings and a meta-analysis have shown that it is associated with essential hypertension [59, 60]; however, other studies failed to reproduce such results [9, 50, 61]. Although we observed that boys with *AGTR1* AC genotype had significantly higher SBP compared to AA carriers, and SBP was much higher among boys with *AGTR1* CC genotype, but the differences did not reach statistical significance due to the small number of homozygotes.

The current study has several limitations. In the present study, BP readings were obtained by using an automatic oscillometric monitor that has been clinically validated. This study investigated only the population of 12–15-year-old adolescents of the second largest city and district of Lithuania. Lithuania is characterized by stable and homogenous ethnic structure of the population [62]. According to the 2011 Lithuanian Population Census, Lithuanians made up about 94.4% of the population in Kaunas County (84% in Lithuania) [63]. The study subjects were randomly selected, so study sample included mostly ethnic Lithuanians.

Furthermore, selection bias, information bias, and confounding factors may affect the results of an observational study [64]. Therefore, further studies are needed to examine a larger population of younger children and older adolescents. There are no measurements of plasma ACE or AGT levels available to correlate directly with the genetic polymorphisms investigated in this study. Pubertal status and biochemical parameters were not evaluated in our study.

## Conclusions

We found that the D allele of the ID polymorphism in the *ACE* gene was significantly associated with hypertension and with obesity-related traits in boys, but not in girls. The evaluated polymorphisms of the *AGT* and *AGTR1* genes did not contribute to the presence of HBP in the present study and may be seen as predisposing factors.

## Abbreviations

ACE: Angiotensin-converting enzyme
AGT: Angiotensinogen
AGTR1: Angiotensin II receptor 1
aOR: Adjusted odds ratio
BMI: Body mass index
BP: Blood pressure
CI: Confidence interval
DBP: Diastolic blood pressure
OR: Odds ratio
SBP: Systolic blood pressure
SD: Standard deviation
WC: Waist circumference

## References

[1] Assadi F (2012). The growing epidemic of hypertension among children and adolescents: a challenging road ahead. Pediatr Cardiol.

[2] Kaplan İ, Sancaktar E, Ece A, Şen V, Tekkeşin N, Basarali MK (2014). Gene polymorphisms of adducin GLY460TRP, ACE I/D, AND AGT M235T in pediatric hypertension patients. Med Sci Monit.

[3] Dulskiene V, Kuciene R, Medzioniene J, Benetis R (2014). Association between obesity and high blood pressure among Lithuanian adolescents: a cross-sectional study. Ital J Pediatr.

[4] Tamosiunas A, Luksiene D, Baceviciene M, Bernotiene G, Radisauskas R, Malinauskiene V (2014). Health factors and risk of all-cause, cardiovascular, and coronary heart disease mortality: findings from the MONICA and HAPIEE studies in Lithuania. PLoS One.

[5] Reklaitiene R, Tamosiunas A, Virviciute D, Baceviciene M, Luksiene D (2012). Trends in prevalence, awareness, treatment, and control of hypertension, and the risk of mortality among middle-aged Lithuanian urban population in 1983-2009. BMC Cardiovasc Disord.

[6] Zaborskis A, Petrauskiene A, Gradeckiene S, Vaitkaitiene E, Bartasiūte V (2003). Overweight and increased blood pressure in preschool-aged children. Medicina (Kaunas).

[7] Grabauskas V, Klumbiene J, Petkeviciene J, Petrauskiene A, Tamosiūnas A, Kriaucioniene V (2008). Risk factors for noncommunicable diseases in Lithuanian rural population: CINDI survey 2007. Medicina (Kaunas).

[8] Chen X, Wang Y (2008). Tracking of blood pressure from childhood to adulthood: a systematic review and meta-regression analysis. Circulation.

[9] Petkeviciene J, Klumbiene J, Simonyte S, Ceponiene I, Jureniene K, Kriaucioniene V (2014). V. Physical, behavioural and genetic predictors of adult hypertension: the findings of the Kaunas Cardiovascular Risk Cohort study. PLoS One.

[10] Klumbiene J, Sileikiene L, Milasauskiene Z, Zaborskis A, Shatckute A (2000). The relationship of childhood to adult blood pressure: longitudinal study of juvenile hypertension in Lithuania. J Hypertens.

[11] Chobanian AV, Bakris GL, Black HR, Cushman WC, Green LA, Izzo JL (2003). The seventh report of the joint National Committee on prevention, detection, evaluation, and treatment of high blood pressure: the JNC 7 report. JAMA.

[12] Brady TM, Fivush B, Flynn JT, Parekh R (2008). Ability of blood pressure to predict left ventricular hypertrophy in children with primary hypertension. J Pediatr.

[13] Sorof JM, Alexandrov AV, Garami Z, Turner JL, Grafe RE, Lai D (2003). Carotid ultrasonography for detection of vascular abnormalities in hypertensive children. Pediatr Nephrol.

[14] Williams SS (2007). Advances in genetic hypertension. Curr Opin Pediatr.

[15] Fava C, Philippe B, Almgren P, Groop L, Hulthén UL, Melander O (2004). Heritability of ambulatory and office blood pressure phenotypes in Swedish families. J of Hyperten.

[16] Kupper N, Willemsen G, Riese H, Posthuma D, Boomsma DI, de Geus EJ (2005). Heritability of daytime ambulatory blood pressure in an extended twin design. Hypertension.

[17] Poch E1, González D, Giner V, Bragulat E, Coca A, de La Sierra A (2001). Molecular Basis of Salt Sensitivity in Human Hypertension. Hyperten.

[18] Beevers G, Lip GY, O'Brien E (2001). ABC of hypertension: the pathophysiology of hypertension. BMJ.

[19] Yazdanpanah M, Aulchenko YS, Hofman A, Janssen JA, Sayed-Tabatabaei FA, van Schaik RH (2007). Effects of the renin-angiotensin system genes and salt sensitivity genes on blood pressure and atherosclerosis in the total population and patients with type 2 diabetes. Diabetes.

[20] Luft FC (2001). Twins in cardiovascular genetic research. Hypertension.

[21] Singh M, Mensah GA, Bakris G (2010). Pathogenesis and clinical physiology of hypertension. Cardiol Clin.

[22] Xue H, Lu Z, Tang WL, Pang LW, Wang GM, Wong GW (2015). First-line drugs inhibiting the renin angiotensin system versus other first-line antihypertensive drug classes for hypertension. Cochrane Database Syst Rev.

[23] Jeunemaitre X, Soubrier F, Kotelevtsev YV, Lifton RP, Williams CS, Charru A (1992). Molecular basis of human hypertension: role of angiotensinogen. Cell.

[24] Lele RD (2004). Hypertension: molecular approach. J Assoc Physicians India.

[25] Mondry A, Loh M, Liu P, Zhu AL, Nagel M (2005). Polymorphisms of the insertion / deletion ACE and M235T AGT genes and hypertension: surprising new findings and meta-analysis of data. BMC Nephrol.

[26] Nantel P, René de Cotret P (2010). The evolution of angiotensin blockade in the management of cardiovascular disease. Can J Cardiol.

[27] Chaves FJ, Pascual JM, Rovira E, Armengod ME, Redon J (2001). Angiotensin II AT1 receptor gene polymorphism and microalbuminuria in essential hypertension. Am J Hypertens.

[28] Bonnardeaux A, Davies E, Jeunemaitre X, Féry I, Charru A, Clauser E, Tiret L, Cambien F, Corvol P, Soubrier F (1994). Angiotensin II type 1 receptor gene polymorphisms in human essential hypertension. Hypertension.

[29] Wang WY, Zee RY, Morris BJ (1997). Association of angiotensin II type 1 receptor gene polymorphism with essential hypertension. Clin Genet.

[30] National High Blood Pressure Education Program Working Group on High Blood Pressure in Children and Adolescents. The fourth report on the diagnosis, evaluation, and treatment of high blood pressure in children and adolescents. Pediatrics. 2004;114(2 Suppl 4th Report):555–576. 15286277

[31] Cole TJ, Bellizzi MC, Flegal KM, Dietz WH (2000). Establishing a standard definition for child overweight and obesity worldwide: international survey. BMJ.

[32] Fernández JR, Redden DT, Pietrobelli A, Allison DB (2004). Waist circumference percentiles in nationally representative samples of African-American, European-American, and Mexican-American children and adolescents. J Pediatr.

[33] Mayer NJ, Forsynth A, Kantachuvesiri S, Mullins JJ, Fleming S (2002). Association of the D allele of the angiotensin I converting enzyme polymorphism with malignant vascular injury. J Clin Pathol Mol Pathol.

[34] Ramos E, Barros H (2005). Prevalence of hypertension in 13-year-old adolescents in Porto. Portugal Rev Port Cardiol.

[35] McNiece KL, Poffenbarger TS, Turner JL, Franco KD, Sorof JM, Portman RJ (2007). Prevalence of hypertension and pre-hypertension among adolescents. J Pediatr.

[36] Mehdad S, Hamrani A, El Kari K, El Hamdouchi A, El Mzibri M, Barkat A (2013). Prevalence of elevated blood pressure and its relationship with fat mass, body mass index and waist circumference among a group of Moroccan overweight adolescents. Obes Res Clin Pract.

[37] Brug J, van Stralen MM, Te Velde SJ, Chinapaw MJ, De Bourdeaudhuij I, Lien N (2012). Differences in weight status and energy-balance related behaviors among schoolchildren across Europe: the ENERGY-project. PLoS One.

[38] Smetanina N, Albaviciute E, Babinska V, Karinauskiene L, Albertsson-Wikland K, Petrauskiene A (2015). Prevalence of overweight/obesity in relation to dietary habits and lifestyle among 7-17 years old children and adolescents in Lithuania. BMC Public Health.

[39] Kunešová M, Vignerová J, Pařízková J, Procházka B, Braunerová R, Riedlová J (2011). Long-term changes in prevalence of overweight and obesity in Czech 7-year-old children: evaluation of different cut-off criteria of childhood obesity. Obes Rev.

[40] Valdés Pizarro J, Royo-Bordonada MA (2012). Prevalence of childhood obesity in Spain: National Health Survey 2006-2007. Nutr Hosp.

[41] Bac A, Woźniacka R, Matusik S, Golec J, Golec E (2012). Prevalence of overweight and obesity in children aged 6-13 years-alarming increase in obesity in Cracow. Poland Eur J Pediatr.

[42] Wirix AJ, Verheul J, Groothoff JW, Nauta J, Chinapaw MJ, Kist-van Holthe JE (2017). Screening, diagnosis and treatment of hypertension in obese children: an international policy comparison. J Nephrol.

[43] Lu X, Shi P, Luo CY, Zhou YF, Yu HT, Guo CY (2013). Prevalence of hypertension in overweight and obese children from a large school-based population in shanghai. China BMC Public Health.

[44] Sorof JM, Lai D, Turner J, Poffenbarger T, Portman RJ (2004). Overweight, ethnicity, and the prevalence of hypertension in school-aged children. Pediatrics.

[45] Field AE, Cook NR, Gillman MW (2005). Weight status in childhood as a predictor of becoming overweight or hypertensive in early adulthood. Obes Res.

[46] Juhola J, Oikonen M, Magnussen CG, Mikkilä V, Siitonen N, Jokinen E (2012). Childhood physical, environmental, and genetic predictors of adult hypertension: the cardiovascular risk in young Finns study. Circulation.

[47] Sethi AA, Nordestgaard BG, Agerholm-Larsen B, Frandsen E, Jensen G (2001). Tybjaerg-Hansen. Angiotensinogen polymorphisms and elevated blood pressure in the general population: the Copenhagen City heart study. Hypertension.

[48] Inoue I, Nakajima T, Williams CS, Quackenbush J, Puryear R, Powers M (1997). A nucleotide substitution in the promoter of human angiotensinogen is associated with essential hypertension and effects basal transcription in vitro. J Clin Invest.

[49] Mattace-Raso FUS, Sie MPS, van der Cammen TJM, Safar ME, Hofman A, van Duijn CM, Witteman JCM (2007). Insertion/deletion gene polymorphism of the angiotensin-converting enzyme and blood pressure changes in older adults. The Rotterdam study. J Human Hyperten.

[50] Papp F, Friedman AL, Bereczki C, Haszon I, Kiss E, Endreffy E (2003). Renin-angiotensin gene polymorphism in children with uremia and essential hypertension. Pediatr Nephrol.

[51] Lemes VA, Neves AL, Guazzelli IC, Frazzatto E, Nicolau C, Corrêa-Giannella ML (2013). Angiotensin converting enzyme insertion/deletion polymorphism is associated with increased adiposity and blood pressure in obese children and adolescents. Gene.

[52] Dhanachandra Singh K, Jajodia A, Kaur H, Kukreti R, Karthikeyan M (2014). Gender specific association of RAS gene polymorphism with essential hypertension: a case-control study. Biomed Res Int.

[53] Fornage M, Amos CI, Kardia S, Sing CF, Turner ST, Boerwinkle E (1998). Variation in the region of the angiotensin-converting enzyme gene influences interindividual differences in blood pressure levels in young white males. Circulation.

[54] O'Donnell CJ, Lindpaintner K, Larson MG, Rao VS, Ordovas JM, Schaefer EJ (1998). Evidence for association and genetic linkage of the angiotensin-converting enzyme locus with hypertension and blood pressure in men but not women in the Framingham heart study. Circulation.

[55] Di Pasquale P, Cannizzaro S, Paterna S (2004). Does angiotensin-converting enzyme gene polymorphism affect blood pressure? Findings after 6 years of follow-up in healthy subjects. Eur J Heart Fail.

[56] Hilliard LM, Sampson AK, Brown RD, Denton KM (2013). The “his and hers” of the renin-angiotensin system. Curr Hypertens Rep.

[57] Grady D, Rubin SM, Petitti DB, Fox CS, Black D, Ettinger B (1992). Hormone therapy to prevent disease and prolong life in postmenopausal women. Ann Intern Med.

[58] Bonnardeaux A, Davies E, Jeunemaitre X, Féry I, Charru A, Clauser E (1994). Angiotensin II type 1 receptor gene polymorphisms in human essential hypertension. Hypertension.

[59] Wang JL, Xue L, Hao PP, Xu F, Chen YG, Zhang Y (2010). Angiotensin II type 1 receptor gene A1166C polymorphism and essential hypertension in Chinese: a meta-analysis. J Renin-Angiotensin-Aldosterone Syst.

[60] Mehri S, Mahjoub S, Hammami S, Zaroui A, Frih A, Betbout F (2012). Renin-angiotensin system polymorphisms in relation to hypertension status and obesity in a Tunisian population. Mol Biol Rep.

[61] Sugimoto K, Katsuya T, Ohkubo T, Hozawa A, Yamamoto K, Matsuo A (2004). Association between angiotensin II type 1 receptor gene polymorphism and essential hypertension: the Ohasama study. Hypertens Res.

[62] Minority Rights Group International, World directory of minorities and indigenous peoples - Lithuania, 2007, available at: http://www.refworld.org/docid/4954ce4f23.html. [Accessed 10 Aug 2017].

[63] Lithuania S (2012). Lithuanian 2011 population census in brief.

[64] Grimes DA, Schulz KF (2002). Bias and causal associations in observational research. Lancet.

